# Latino Sexual Minority Men Living with HIV in South Florida have Varied Experiences of Intersectional Discrimination: A Mixed Methods Pilot Study

**DOI:** 10.1007/s10461-025-04691-1

**Published:** 2025-03-24

**Authors:** Emily M. Cherenack, Jaislene Viñas, Sol Fernandez-Nocito, Jennifer V. Chavez, Favour Ebiala, Omar Valentin, Joseph P. De Santis

**Affiliations:** 1 University of Miami, Miami, FL USA; 2 Florida International University, Miami, FL USA

**Keywords:** Discrimination, Minority stress, Sexual minority, Latino, Stigma, HIV

## Abstract

Culturally tailored behavioral interventions are needed to improve HIV treatment outcomes among Latino gay, bisexual, and other sexual minority cisgender men (LSMM) living with HIV. From 2022 to 2023, this study collected cross-sectional survey data (*n* = 58) and qualitative interview data (*n* = 10) to describe intersectional discrimination and obtain insights for tailoring interventions to address discrimination among LSMM living with HIV in Miami, Florida, USA. The sample was diverse in age (range 21–75), sexual orientation (83% gay, 17% bisexual), and country of origin (71% born outside the USA), with many participants born in Cuba (28%), and more than half of participants (64%) completing the study in Spanish. Experiences of discrimination varied, with 41% personally experiencing discrimination in the past year. Over one-third reported experiences of violence due to discrimination (36% physically attacked, 35% sexually assaulted). Lifetime discrimination was most often attributed to sexual orientation (60%). All forms of discrimination were more severe among men from minoritized racial groups, and some forms of discrimination varied by time spent living in the USA. In interviews, discrimination was described as less severe in the USA compared to countries of origin, driven in part by religiosity and machismo. The impacts of discrimination ranged from mild and temporary to traumatic and persistent. Intervention suggestions included focusing on broad stressors, offering group- and individual options, prioritizing in-person interventions, offering trauma-informed care, and providing legal and immigration services. Findings demonstrate the need for multiple interventions to meet the varied needs, experiences, and preferences of LSMM living with HIV.

## Introduction

Latino gay, bisexual, and other sexual minority cisgender men (LSMM) are a priority population for HIV prevention and treatment in South Florida, which contains the metropolitan area with the highest rate of HIV diagnoses in the United States (USA) [1]. In 2022, Latino cisgender men made up the largest proportion (32%) of adults diagnosed with HIV in Florida, with the number of diagnoses among individuals from Cuba increasing over time [1]. An estimated 27% of LSMM with HIV in Florida are not virally suppressed [2], with viral suppression rates lower among Latino adults than non-Latino White and Black adults [1]. This indicates a need for tailored interventions to improve HIV outcomes among LSMM with HIV.

LSMM with HIV in the USA may face unique stressors that elevate risk for disparities along the HIV care continuum. LSMM with HIV may experience discrimination, such as homonegativity, HIV stigma, or ethnic discrimination, directed towards multiple intersecting identities [3–7]. The chronic stress caused by marginalization of intersectional identities is defined as “intersectional minority stress” and has been associated with poor health [3, 4]. Mental health stigma and Latino cultural norms that promote aggressive masculinity, known as “machismo,” may present barriers to seeking medical and mental health care, and norms around strong commitment to family, known as “familismo,” may lead men to fear disclosing their sexual orientation and HIV status to family [5, 8–14]. Stigma, trauma, and low family and other social support among Latino immigrants have been identified as barriers to HIV care in qualitative research [8].

Current behavioral health interventions require translation and cultural adaptation to be delivered in English and Spanish and address the distinct psychosocial experiences of LSMM with HIV. Behavioral interventions that fail to account for the difficulty of engaging in healthy behaviors in the context of social marginalization may increase self-blame and shame, a strong predictor of immune dysregulation [15–18]. Pilot research among LSMM with HIV in Los Angeles, California has provided preliminary evidence for a potential emotional benefit of cognitive-behavioral therapy focused on coping with discrimination [19]. However, most Latino individuals in Los Angeles and in the aforementioned pilot study were of Mexican descent [19]. Latino individuals in Miami are more often from Caribbean and South American countries and may have different needs due to unique historical contexts and cultural influences [20].

At the time this study was implemented, an increase in immigration, migration, and asylum seeking among individuals from South and Central America became part of public discourse in news media and politics [21, 22]. In 2022, there was legislation passed in Florida limiting discussion of sexual orientation in schools, “The Parental Rights in Education Act (HB 1557),” which was labeled as the “Don’t Say Gay Law.” Research among sexual minority parents shows there was concern about this law furthering anti-sexual minority sentiment [23], but less is known about how the law impacted individuals who were not involved in the school system. Consequently, this study can provide unique insight into discrimination at a time when political events in Florida and the USA included a focus on sexual minorities and immigration and asylum seeking.

The purpose of this mixed-methods observational study was to provide a quantitative and qualitative description of sexual minority stress, HIV stigma, and intersectional discrimination among LSMM with HIV in Miami, Florida. Aims included (a) describing discrimination, sexual minority stress, and HIV stigma among LSMM with HIV via surveys and qualitative interviews; (b) comparing discrimination, sexual minority stress, and HIV stigma across sociodemographic characteristics; and (c) identifying areas for culturally tailoring minority stress coping interventions for LSMM with HIV, especially men of South American, Central American, and Caribbean descent.

## Methods

### Study Design

This analysis includes cross-sectional survey and in-depth interview data collected as part of “Siempre Contigo (“Always with You),” a convergent mixed-methods pilot study that assessed stress, coping, and immune regulation to tailor behavioral health interventions for LSMM with HIV. The current analysis focuses on descriptions of discrimination, stigma, and minority stress.

### Setting

This study took place in Miami-Dade County, Florida from August 2022 to September 2023. In 2022, it was estimated that 69% of people living in Miami-Dade County were Hispanic or Latino, and 54% of residents were born outside the US, with the most common origins being Cuba, Haiti (not a Spanish-speaking country), and Colombia [20].

### Participants

Eligibility criteria included being Latino or Hispanic, identifying as a cisgender man, previously being diagnosed with HIV, being at least 18 years-of-age, being willing to travel to Miami for an in-person visit, being able to communicate in Spanish or English, and either identifying as gay or bisexual (regardless of recent sexual behavior) or identifying as a sexual minority other than gay/bisexual (e.g., other/not listed) and having any type of anal sex with a man in the past year. Men who identified as straight/heterosexual were excluded. A subset of enrolled participants who reported personally experiencing any stigma or discrimination in the past year on the survey were invited to complete an in-depth qualitative interview.

Recruitment methods included contacting men in consent-to-contact databases, handing out flyers at community events, posting flyers in HIV and infectious disease clinics, offering flyers to participants attending visits for other research studies, and posting the study on the university research website. Flyers and other materials were designed in collaboration with a community advisory board including Latino and Hispanic individuals and referenced a study on stress and coping among Latino men.

### Procedures

All procedures were offered in Spanish and English. Candidates could complete an eligibility screener online or with a study team member. After completing an informed consent process, participants were invited to complete a study visit, which included a psychosocial survey and a blood draw. In-depth qualitative interviews lasted about one hour per participant and were conducted via video conference. Study team members interacting with participants included two bilingual staff of Latino descent who were trained and supervised by the lead author, a PhD-level researcher trained in global mixed methods research. Interviews followed a semi-structured guide with example questions and prompts and were audio recorded. Participants were compensated for their time.

### Ethics

This study was approved by the University of Miami IRB. All participants provided written informed consent.

### Measures

Sources of data included the survey, in-depth semi-structured interview transcripts, and the blood test for HIV viral load. Measures previously validated in English and Spanish were used whenever possible. Other measures were translated and back-translated from English to Spanish and pretested by bilingual staff.

#### Sociodemographics

Sociodemographic characteristics were measured using items from the Center for Latino Health Research Opportunities demographics form, which included questions on race, immigration, education, employment, income, financial concerns, country of birth, and health insurance status. A Brief Acculturation Scale for Hispanics asked participants what languages they use to speak, read, and think, which was operationalized into *low* or *high* language-based acculturation [24]. HIV viral load was measured in blood and defined as undetectable at < 20 copies/mL.

#### Discrimination, Stigma, and Minority Stress

The Intersectional Discrimination Index [25] included five sub-scales/indices for anticipated and enacted intersectional discrimination: (1) day-to-day discrimination over the lifetime, (2) day-to-day discrimination over the past year, (3) major discrimination over the lifetime, (4) anticipated discrimination, and (5) attributions for discrimination. The Intersectional Discrimination Index [25] questionnaire led with the following introduction: “The questions are about experiences related to who you are. This includes both how you describe yourself and how others might describe you. For example, your race, ethnicity, HIV status, skin color, ancestry, nationality, religion, gender, sexuality, age, weight, disability or mental health issue, and income.” Day-to-day and major discrimination were measured using checklists/indices, without an expectation of internal consistency [25].

The nine-item Intersectional Day-to-Day Discrimination Index [25] asked participants how often they experienced daily instances of discrimination, such as being called names, being stared at, and being treated as if others are afraid of them. Response options included *Never*; *Yes*,* but not in the past year*; *Yes*,* once or twice in the past year*; and *Yes*,* many times in the past year*. If participants completed at least 80% of items, missing items were imputed as *Never*. For a measure of lifetime day-to-day discrimination, the number of non-zero (i.e., ever) responses were summed so that higher scores indicated a greater number of types of daily discrimination experienced across the lifetime (range 0–9). For a measure of past-year day-to-day discrimination, items for discrimination over the past year were summed for an overall score where higher scores indicated more frequent day-to-day discrimination over the past year (range 0–18).

The Intersectional Major Discrimination Index [25] asked participants how often they had experienced major instances of discrimination across their lifetime, such as being refused healthcare, being physically attacked, and being harassed. Response options included *Never (0)*, *Once (1)*, and *More than once (2)*. If participants completed at least 80% of items, missing items were imputed as “*Never*,” and responses were summed with higher scores indicating more instances of major discrimination across their lifetime (range 0–26).

The Intersectional Anticipated Discrimination Scale [25] asked participants how much they agree, from *Strongly disagree (0)* to *Strongly agree (4)*, with worries about discrimination such as “I expect to be pointed at, called names, or harassed when in public.” For participants completing at least 80% of the items, a mean score was calculated, where higher scores indicated higher anticipated discrimination. Internal reliability was high (Cronbach’s alpha = 0.9) and similar to prior research on a larger sample, where Cronbach’s alpha = 0.9 [25].

To measure attributions for intersectional discrimination, participants were asked “Thinking of all of the times that you have been treated unfairly or poorly because of who you are, how often do you think each of the following was a reason why others treated you this way?” Response options included ethnicity, race, gender, HIV status, sexual orientation, weight, income/employment, other, and not applicable.

Separate measures were used to assess sexual minority stress and HIV stigma. A modified nine-item CARS Sexual Minority Stress Subscale [26] measured sexual minority stress by including the original five sexual minority stress items, in addition to four non-specific minority stress items from the CARS adapted to focus on sexual minority stress. Example items include “I was rejected by a family member or friend after telling him/her my sexual orientation” and “People treat me unfairly because of my sexual identity.” Each item was rated on a scale from *Strongly disagree (1)* to *Strongly agree (6)*. Scores were calculated by summing the nine items, with one item reverse scored, so that higher scores (range 9–54) indicate higher sexual minority stress. Internal consistency for sexual minority stress was acceptable (Cronbach’s alpha = 0.8), and similar to internal consistency scores for the original measure in prior research (e.g., 0.7) [27].

A 12-item HIV Stigma Scale [28] measured agreement with statements such as “I have lost friends by telling them I have HIV” and “people with HIV are treated like outcasts.” Responses were summed so higher scores reflect higher HIV-related stigma across domains of personalized stigma, disclosure concerns, concerns with public attitudes, and negative self-image (range 12–48) [28]. Internal consistency for HIV-related stigma was high (Cronbach’s alpha = 0.9).

Lastly, participants were asked if they had personally experienced unfair treatment or discrimination, knew others who had experienced unfair treatment or discrimination, or saw discrimination directed towards their community or people like them in the past year, with binary (yes/no) response options.

### Qualitative Interview Guide

The interview guide was designed to capture participants’ descriptions of their identifies, of positive and negative experiences related to these identities, and experiences of discrimination. Participants were also asked their opinions on hypothetical programs to improve coping with discrimination. Questions and example prompts are included in Table 1. As part of the aims of the larger study, additional interview questions focused on coping with general stress; however, these items were only included as part of the current analysis if they contained themes pertinent to discrimination.

**Table 1:** Interview guide excerpts

Introduction: People have unique combinations of personal identities based on their sense of who they are, their personal and family history, the communities they feel connected to, and how other people view them. For example, people may have multiple identities associated with their race, ethnicity, gender, sexual orientation, country of origin, religion, HIV status, or job, among many other things.
- What identities do you use to describe yourself?
- What has your experience been having multiple identities, such as being _____ and _____?
- Can you describe any positive experiences related to your identities?
o What do you like about being _____ and _____?
- Can you describe any stressful experiences related to your identities?
o What has been your experience with racism, homophobia, HIV stigma or other forms of discrimination?
o How do experiences of discrimination compare to other stressors in your life?
- We would like your opinion on whether gay/bisexual Latino men living with HIV would be interested in a program to improve coping skills to manage stress related to stigma and discrimination. For example, a program could involve having weekly meetings with a coach and practicing coping skills in daily life.
o What would lead men to be more or less interested in taking part in a program to improve coping?
o What would be an ideal length, format, number of sessions, type of facilitator?

### Analysis

#### Quantitative

Descriptive statistics were used to characterize the sample in terms of sociodemographics and intersectional discrimination, sexual minority stress, and HIV stigma. Analysis of Variance tests (ANOVAs), t-tests, and Pearson correlations were used to compare mean discrimination, stigma, and minority stress indicators with each other and across sociodemographic characteristics. Indices of discrimination, stigma, and minority stress were compared across sexual orientation (gay vs. bisexual), time in the USA (born in the USA, in the USA less than 5 years, in the USA 5 or more years), and race. Due to low counts for races other than White, we combined Black, Indigenous, Multi-Racial, and other-identified into a single non-White category to represent historically minoritized groups. Missing data were minimal (< 5% across constructs of interest) and handled using pairwise deletion. Analysis was performed in R Version 4.4.0 [5^29^].

#### Qualitative

Interviews were transcribed using intelligent/edited transcription methods to remove filler words and increase clarity and then translated into English by bilingual individuals. Transcripts were imported into NVivo V14. The lead author and four research staff, of whom three were Latino/Hispanic and bilingual, read the transcripts to increase familiarity with the data and had ongoing discussions regarding the interpretation of themes and potential sources of bias in our interpretations. Using a structural coding approach, the initial set of top-level codes were derived from interview guide questions. Qualitative description, which summarizes events in the everyday language used by participants to provide straight descriptions and increase validity and interpretability, was used to refine and add additional top-level and nested codes and summarize codes as themes that remain close to surface descriptions in the data [30].

In qualitative research it is important to ensure that data saturation occurred. Data saturation, the subjective determination by the study team that no new data have emerged from the interviews [31], was determined by the first author and confirmed and verified by the last author. Because data saturation was reached, no additional interviews were conducted.

Qualitative research also requires rigor or trustworthiness, the qualitative equivalent of data verification. The authors used two methods of data verification. First, they used peer-review/peer-debriefing. This method of data verification confirms the study’s findings are consistent with previous research [31]. Peer-review/debriefing was achieved by the first author and a research assistant, who reviewed all codes that emerged from the data and resolved any discrepancies in coding through discussions that resulted in a consensus on the final codes. Another method of data verification was the use of thick and rich descriptions in the form of participant quotes [31]. These participant quotes were used to provide detail, context, emotions, etc. which demonstrate that the study’s findings are congruent with previous research [31].

#### Triangulation of Qualitative and Quantitative Results

Qualitative and quantitative analyses were planned a priori, but conducted iteratively, such that for *post hoc* analyses, qualitative interviews were used to generate potential explanations for relationships and inconsistencies found in quantitative data, and quantitative data were used to confirm if themes in the interviews were also represented in the larger quantitative sample.

#### Sample Size

As a descriptive pilot study, the overall sample size was determined by the number of participants that could be recruited in the 1-year study period.

## Results

### Enrollment

Overall, 149 unique screening surveys were completed, which resulted in 83 eligible individuals. The most common reasons for being ineligible were not living with HIV (*n* = 57), not being a sexual minority (*n* = 45), and not being Latino or Hispanic (*n* = 21). Out of 83 eligible men, four declined to participate due to being busy, one was not interested, one declined for unknown reasons, and 19 did not return our calls. A total of 58 participants enrolled in the study, which is 70% of eligible men and 90% of men successfully contacted. Out of 24 men eligible for the interviews, about half (*n* = 13) were able to be reached to complete the in-depth interviews, of whom 10 completed the interview.

### Sociodemographic Characteristics

The sample was diverse in terms of sociodemographic characteristics (Table 2). Age ranged from 21 to 75, with a mean of 49. Forty-eight (83%) participants were gay, and 10 (17%) were bisexual. All participants were taking antiretroviral therapy. Based on a cutoff of 20 copies/mL, 14 (24%) had a detectable HIV viral load. Average years since HIV diagnosis was 16 (*SD* = 11), with a range from less than 1 year up to 38 years. While most participants identified their race as White (*n* = 46, 79%), the sample also included participants who were Black (*n* = 6, 10%), Indigenous (*n* = 1, 2%), Multi-Racial (*n* = 1, 2%), and who wrote in “Latino” or “African” for “other” (*n* = 2, 3%). Forty-one (71%) participants were born outside of the incorporated USA, 39 (67%) were USA citizens, and 9 (16%) lived in the USA fewer than 5 years. Cuba (*n* = 16, 28%) was the most common country of birth outside of the USA. Regarding language, 37 (64%) preferred to complete the study in Spanish. Low language-based acculturation was found among 9 of 9 (100%) participants residing in the USA for less than 5 years, 27 of 30 (90%) participants residing in the USA more than 5 years but not born in the USA, and 7 of 19 (37%) of participants born in the USA. Interviews clarified that some participants were born in the USA but raised elsewhere.

**Table 2:** Sample demographics among 58 Latino sexual minority men living with HIV in Miami, Florida

	Mean (SD) or *n* (%)
Age	49 (± 14)
Race^1^	
White	46 (79)
Black	6 (10)
Other	2 (3)
Indigenous	1 (2)
Multi-Racial	1 (2)
Sexual orientation is Gay (vs. Bisexual)	48 (83)
Monthly income per dependent, including self^1^	1300 (± 970)
Preferred language is English	21 (36)
Country/territory of birth	
USA (Exclusive of Puerto Rico)	17 (29)
Cuba	16 (28)
Colombia	5 (9)
Puerto Rico	4 (7)
Nicaragua	4 (7)
Venezuela	3 (5)
Ecuador	2 (3)
Honduras	2 (3)
Peru	2 (3)
Brazil	1 (2)
El Salvador	1 (2)
Other	1 (2)
Citizenship status^2^	
USA citizen	39 (67)
Permanent resident	6 (10)
Without papers or expired visa	4 (7)
Student/tourist visa, or temporary resident	4 (7)
Asylum	4 (7)
Years living in USA	
Lifetime^3^	19 (33)
5 or more years	30 (52)
Less than 5 years	9 (16)

Percentages rounded to the nearest whole number. ^1^*n* missing = 2. ^2^*n* missing = 1. ^3^Some participants born in Puerto Rico reported being born in the USA and were not asked a follow-up question on years spent in the USA

### Discrimination, Sexual Minority Stress, and HIV Stigma

Discrimination outcomes are presented in Table 3. Based on self-report, over the past year, 24 (41%) participants personally experienced discrimination, 17 (29%) knew someone that experienced discrimination, and 22 (38%) saw discrimination directed towards their community or others like them. Sixteen (28%) did not experience or see anyone else experience unfair treatment or discrimination. Participants attributed lifetime experiences of discrimination most often to sexual orientation (*n* = 35, 60%), followed by ethnicity (*n* = 22, 38%), HIV status (*n* = 16, 28%), race (*n* = 14, 24%), gender (*n* = 12, 21%), weight (*n* = 9, 16%), and income (*n* = 7, 12%). After excluding participants who reported none (*n* = 17), the most common combinations of attributions were reporting discrimination due to sexual orientation only (*n* = 4) and reporting discrimination due to ethnicity, race, and sexual orientation (*n* = 4). On average, participants reported 2 attributions.

**Table 3:** Minority stress, stigma, and discrimination among 58 Latino sexual minority men living with HIV in Miami, Florida

	Mean (SD) or *n* (%)
Discrimination in the past year	
I have not experienced or witnessed discrimination	16 (28)
I have personally experienced discrimination	24 (41)
Someone I know has experienced discrimination	17 (29)
Discrimination has been directed towards my community or others like me	22 (38)
Lifetime day-to-day discrimination (scale range 0–9)	4.1 (± 3.0)
Past-year day-to-day discrimination (scale range 0–18)	3.7 (± 4.7)
Anticipated discrimination (scale range 0–4)	1.4 (± 1.0)
Lifetime major discrimination (scale range 0–26)	4.5 (± 5.0)
Sexual minority stress (9–54)	27 (± 9.4)
HIV stigma (scale range: 12–48)	28 (± 8.3)
*Missing*	2 (3)
Lifetime discrimination attributions	
Sexual orientation	35 (60)
Ethnicity	22 (38)
HIV status	16 (28)
Race	14 (24)
Gender	12 (21)
Weight	9 (16)
Other	8 (14)
Income	7 (12)

The Intersectional Discrimination Index [25] was used to measure day-to-day discrimination over the lifetime, day-to-day discrimination over the past year, major discrimination over the lifetime, anticipated discrimination, and attributions for discrimination. The CARS Sexual Minority Stress Subscale [26] measured sexual minority stress. A 12-item HIV Stigma Scale [28] measured HIV stigma

The most common forms of major discrimination across the lifetime were losing a close relationship (*n* = 28, 48%), being threatened with a physical or sexual attack (*n* = 21, 36%), being physically attacked (*n* = 21, 36%), and being sexually assaulted (*n* = 21, 35%). These were followed by being repeatedly harassed (*n* = 17, 29%), being fired or not hired for a job (*n* = 15, 26%), being unreasonably stopped by the police (*n* = 13, 22%), having to move (*n* = 9, 16%), having property damages/stolen/vandalized (*n* = 8, 14%), being evicted or denied housing (*n* = 5, 9%), being refused healthcare (*n* = 5, 9%), being unable to open a bank account/get a loan/cash a check (*n* = 4, 7%), and being expelled or suspended from school (*n* = 3, 5%).

### Correlations Between Discrimination, Stigma, and Minority Stress

All discrimination and stigma scales correlated positively from *r* = 0.43 to 0.78 (Table 4). The lifetime day-to-day discrimination, anticipated discrimination, HIV stigma, and sexual minority stress scales were split into low/medium/elevated categories based on the 25th and 75th percentiles. There were 44 total combinations represented, with 45 (76%) participants having a mix of low/medium/elevated, 7 (12%) low on all five measures, 1 (2%) medium on all measures, and 5 (9%) elevated on all measures.

**Table 4:** Pearson correlations for discrimination, minority stress, and stigma measures

	Day-to-day past-year discrimination	Day-to-day lifetime discrimination	Anticipated discrimination	Major discrimination	Sexual minority stress
Day-to-day lifetime discrimination	0.78*				
Anticipated discrimination	0.45*	0.44*			
Major discrimination	0.66*	0.69*	0.48*		
Sexual minority stress	0.58*	0.62*	0.59*	0.55*	
HIV stigma	0.49*	0.62*	0.66*	0.43*	0.73*

*p < 0.05. The Intersectional Discrimination Index [25] was used to measure day-to-day discrimination over the lifetime, day-to-day discrimination over the past year, major discrimination over the lifetime, anticipated discrimination, and attributions for discrimination. The CARS Sexual Minority Stress Subscale [26] measured sexual minority stress. A 12-item HIV Stigma Scale [28] measured HIV stigma

### Discrimination, Sexual Minority Stress, and HIV Stigma across Sociodemographic Characteristics

Indices of discrimination, stigma, and minority stress were compared across sociodemographic characteristics. There were no significant differences by sexual orientation (*ns*), but race and time in the USA were associated with multiple indices of discrimination (Table 5). Men in the USA for less than 5 years and men born in the USA had higher lifetime and past-year day-to-day discrimination compared to men in the USA for more than 5 years. Men in the USA less than 5 years also had higher major discrimination compared to men born in the USA and men in the USA for more than 5 years. Compared to men from minoritized racial groups, White participants showed lower lifetime day-to-day discrimination, past-year day-to-day discrimination, anticipated discrimination, major discrimination, sexual minority stress, and HIV stigma.

**Table 5:** Intersectional discrimination, sexual minority stress, and HIV stigma by time in the United States and race (*N* = 58)

	Time in United States (Mean, SD)				
	Born in USA (*n* = 19)	5 + Years (*n* = 30)	< 5 Years (*n* = 9)	F value	P-value
Lifetime day-to-day discrimination	5 (± 3)	3 (± 2)	6 (± 3)	7.7	0.001*
Past-year day-to-day discrimination	6 (± 6)	2 (± 2)	6 (± 5)	6.2	0.004*
Anticipated discrimination	2 (± 1)	1 (± 1)	2 (± 1)	1.2	0.324
Lifetime major discrimination	6 (± 5)	3 (± 3)	8 (± 8)	5.2	0.009*
Sexual minority stress	30 (± 12)	24 (± 7)	28 (± 7)	2.3	0.114
HIV stigma	29 (± 10)	26 (± 7)	29 (± 7)	0.9	0.394

	Race (Mean, SD)			
	Non-white (*n* = 10)	White (*n* = 46)	F value	P-value
Lifetime day-to-day discrimination	6 (± 3)	4 (± 3)	6.7	0.012*
Past-year day-to-day discrimination	8 (± 6.7)	3 (± 4)	11.5	0.001*
Anticipated discrimination	2 (± 1)	1 (± 1)	5.0	0.030*
Lifetime major discrimination	8 (± 7)	4 (± 4)	7.6	0.008*
Sexual minority stress	33 (± 8)	25 (± 9)	6.1	0.017*
HIV stigma	34 (± 8)	26 (± 8)	5.9	0.018*

*p < 0.05. Means and SD rounded to nearest whole value. P-values and F-values are from analysis of variance tests. For all variables, higher values indicate worse discrimination, minority stress, or stigma

### Qualitative Results

#### Qualitative Sample

The qualitative interview sample is described in Table 6, with some details such as age and country of origin generalized to maintain anonymity. Sub-sample demographics for the interviews were similar to the larger study sample (e.g., 79% and 80% were White, and 29% and 30% were born in USA for quantitative and qualitative samples, respectively).

**Table 6:** Qualitative sample description (*N* = 10)

	*n* (%)
Age	
50–60 s	6 (60%)
20–30 s	4 (40%)
Sexual orientation	
Gay	7 (70%)
Bisexual	3 (30%)
Race	
White	8 (80%)
Black	1 (10%)
Other race	1 (10%)
Country of origin	
USA	4 (40%)
Caribbean	5 (50%)
South America	1 (10%)

#### Descriptions of Identities

Participants were asked what identities they used to describe themselves, and which of these identities were most important to their lives. Two participants did not feel aligned with any specific identity.

…just a normal human being. – *P2 (50s/60s*,* Gay*,* White*,* Born in USA)*.

It’s really hard for me to identify myself as something. – *P6 (50s/60s*,* Gay*,* White*,* Born in Caribbean)*.

Some participants noted that although they describe themselves as gay, they do not consider their sexual orientation to be an important part of their identities. One participant noted how his perceptions of being gay changed as he realized he did not have to conform to stereotypes regarding masculinity. This touches on a repeated theme throughout the interviews about the importance of masculinity. One participant stated that he doesn’t identify with the gay community because he attributes so many negative experiences in his life, including acquiring HIV, to being gay.

I’m gay, but that doesn’t describe who I am as a person. *– P8 (20s/30s*,* Gay*,* White*,* Born in Caribbean)*.

I’m myself. I love men, and that’s the only thing. I don’t identify myself in that community. *– P6 (50s/60s*,* Gay*,* White*,* Born in Caribbean)*.

I could take a step back from the situation and be gay without being necessarily feminine or letting my hair grow out. – *P5 (20s/30s*,* Bisexual*,* Race is Other*,* Born in South America)*.

The most common salient identities were being Latino/Hispanic or being from a particular country or territory. Multiple participants identified as both Latino/Hispanic and as American, with one describing a multi-cultural community. This participant also noted the intersection of ethnicity and race, such that being White as a Latino can be unique from being White as a non-Latino.

We’re living in a culture that definitely joins together all of those groups. […] People even tell me ‘Oh you’re [Caribbean nationality]? You look White.’ And yeah, I’m not ‘White-White,’ but I am ‘White.’ – *P7 (50s/60s*,* Gay*,* White*,* Born in Caribbean)*.

#### Positive Experiences Due to Identities

Participants reported positive experiences when they were accepted by others for living with HIV or for being gay.

Other people are like, ‘oh, that’s not an issue,’ even if they’re [HIV] negative, that’s not an issue with them […] so, you know, in some cases have been a positive experience. – *P1 (50s/60s*,* Gay*,* White*,* Born in Caribbean)*.

Multiple participants reported the benefit of speaking two languages and being comfortable in multiple cultures. Participants mentioned unique job or learning opportunities because of being male and American.

Feeling comfortable with the two cultures, American and Hispanic. Being able to interact in both languages. It’s fun. – *P7 (50s/60s*,* Gay*,* White*,* Born in Caribbean)*.

Participants reported enjoying finding like-minded peers and experiencing a sense of “camaraderie” based on shared backgrounds. Three participants from the Caribbean noted pride in their nationality, including pride in their country and in other individuals for succeeding.

The Latin community is breaking down barriers, especially with music and in acting and things like that. So I guess, in that way, I like the fact that I am Latino. *– P8 (20s/30s*,* Gay*,* White*,* Born in USA)*.

#### Discrimination Due to Identities

Participants varied widely in the extent to which they faced discrimination or viewed discrimination as a negative experience. For HIV stigma, some participants did not experience HIV stigma, while another participant reported lasting trauma and anxiety about disclosing his HIV status due to prior rejection. One participant reported HIV stigma was worse in his country of origin, which is one reason he left.

I’m not really stressed over being HIV positive or undetectable or telling other people. -- *P8 (20s/30s*,* Gay*,* White*,* Born in USA)*.

The first negative experience was when I was first diagnosed with the disease, with the virus. That was when I had my first negative experience with discrimination. And from there on out, it was pretty traumatic. First, because I had to keep secret my condition, keep it a secret. And the few people that would find out weren’t too pleasant. There was an immediate rejection. – P4 *(50s/60s*,* Gay*,* White*,* Born in USA)*.

Discrimination based on ethnicity was less common, although one participant commented that South Florida is unique due to the large Latino community, and other states in the USA have not been as friendly to Latino individuals. One individual reported witnessing discrimination in the form of stereotypes expressed by Latino people from different countries towards each other. Another participant described how people who are undocumented are at higher risk for relationship abuse and a lack of opportunities. Multiple participants indicated being criticized or treated differently because of their accents.

They [people without documents] might not be able to get a car or have a license or things of that kind. – *P5 (20s/30s*,* Bisexual*,* Race is Other*,* Born in South America)*.

With respect to discrimination directed towards sexual orientation, most participants who had lived outside of the USA reported that discrimination was less severe in the USA compared to Latin American countries. This was attributed to three factors: (1) machismo, (2) religiosity, and (3) a general sense of acceptance in the USA.

The American is very different to the Latino in that aspect. The American doesn’t care. What they care about is if you’re a good person, if you’re a good citizen, you pay your taxes, and you treat me well. That’s all that matters…[Interviewer: Why is it different? ] First, religion. And second, the culture. The culture of the macho, macho man. It’s a stereotype created by Latino culture. – *P4 (50s/60s*,* Gay*,* White*,* Born in USA)*.

The culture here in the United States is different. In other countries maybe if you look different, act different in any way – you could be discriminated or treated differently or other things. – *P7 (50s/60s*,* Gay*,* White*,* Born in Caribbean)*.

They look at you like a weirdo, like something strange. They try to disrespect you, like the ‘machita’ way of thinking, you know? And in my country is like that, and then here in the Latino community, I don’t deal with that, with the machismo. – *P6 (50s/60s*,* Gay*,* White*,* Born in Caribbean)*.

One participant described how being Latino and being an immigrant increases the risk of anti-gay discrimination.

[As a bisexual, if you are] American, you have your rights, done. But when you are Latino, and you come here, you are doubly discriminated. – P9 *(20s/30s*,* Bisexual*,* Black*,* Born in Caribbean)*.

Another participant described feeling more vulnerable to negative experiences due to being Latino and gay, even if he hasn’t experienced them recently. This participant also indicated being bothered when seeing discrimination in news or movies.

Multiple older participants reported that anti-gay discrimination has decreased over time, both in society overall, which was partially attributed to increased representation in the media, and among their family members and acquaintances. Participants reported previously being rejected by family members and neighbors and having since reconciled these relationships.

They learn, thank God. And they realize there’s nothing different. Sexual preference is the only difference. So, it’s just a much nicer situation in the neighborhood. – *P2 (50s/60s*,* Gay*,* White*,* Born USA)*.

#### Impact of Discrimination

The impact of discrimination ranged from minimal to traumatic. The primary negative impacts were lower self-esteem, feeling hurt, worse depressive symptoms, and stress. One participant described how discrimination only impacts him temporarily.

Discrimination can make you feel stressed out. - *P1 (50s/60s*,* Gay*,* White*,* Born in Caribbean)*.

Usually when I feel things like discrimination, in the moment it can affect me […] that can’t change my whole day or make me feel low all day. – P9 *(20s/30s*,* Bisexual*,* Black*,* Born in Caribbean)*.

Multiple participants reported that other stressors were more important than discrimination, including acting as a caretaker to aging parents, interpersonal conflicts with family, financial hardship, and the general “stress of daily life.”

#### Recommendations for Coping Interventions

Four participants reported a program to help LSMM with HIV cope with discrimination would be helpful. One participant felt it was not helpful for him personally, but it might help others. The other participants felt it depended on the individual, or they did not state an opinion about whether it would be helpful. In terms of content and approach, quotes and suggestions are outlined in Table 7.

Yeah, that’s a good thing. – P6 *(50s/60s*,* Gay*,* White*,* Born in Caribbean)*.

It would probably help a lot of people. I don’t know how much it would help me, but I’m not one to go into a group and tell them all everything about my life. – P1 *(50s/60s*,* Gay*,* White*,* Born in Caribbean)*.

**Table 7:** Suggestions for interventions from qualitative interviews (*N* = 10)

Suggestions for Interventions	Example quote
Content suggestions	
Stress coping (*n* = 1), reducing anger due to stress (*n* = 1), and problem-solving (*n* = 3).	Managing stress by the meditation, yoga, whatever practice of wellness, like walking, yoga, meditation, [would] help 100%.
Violence/trauma (*n* = 2)	A lot of people come with very traumatic relationships. The majority come with relationships that are either romantic partners or family. [I: So a program like this? ] Would help a lot.
Immigration services (*n* = 2)	The first thing to be focused on is permitting them to work. Latinos, let’s say here, the majority – the grand, grand majority, don’t want to be gifted anything. We want to earn it.
Legal services (*n* = 1)	I would support talks as well with professionals who could say ‘hey these are your rights, you can do this here, you’re protected under this law, you can sue this person.’
HIV diagnosis (*n* = 1)	When they get diagnosed, they may have heard the most stupid things about it. Like, oh, ‘I’m gonna die, I cannot have sex, nobody’s gonna want me, nobody’s gonna wanna date me’, that kind of stuff. I think at the beginning it’s very important.
Sexuality (*n* = 1)	Sexology as well, because it would help individuals to not feel bad about themselves.
Formatting suggestions	
In-person only *(n* = 2)	A one-on-one and in-person, not via Zoom or FaceTime. Like no, it has to be in person.
Telehealth option (*n* = 3)	I have transportation, so whenever possible, I prefer in person. But if I’m not feeling great or it’s a weird hour, the day in a place that is too far away, then I may prefer Zoom.
Group only (*n* = 4)	In a group because there are already people there, present, that can talk about their experiences.
Individual only (*n* = 1)	It’ll be better individual because I don’t know how gays can be in front of other gays.
Group and individual (*n* = 3)	Individually, because some people might not want to talk yet about the subject, so making it private. And in groups too because some people can do it in groups.
Session frequency 2/Weekly × 4 h (*n* = 1) 1/Weekly (*n* = 2) 2/Monthly × 1.5 h (*n* = 1) 1/Monthly (*n* = 1) Depends (*n* = 2)	It all depends on how long it takes for you to see the change in yourself.

One participant suggested having a weekly program just for men newly diagnosed with HIV (within the last year) to dispel myths about HIV and manage fears of dying among newly diagnosed men. One participant suggested facilitating work approvals for immigrants, while another participant suggested providing legal guidance regarding immigration. Other content suggestions included managing anger and rage; meditation, walking, and yoga for stress management; adapting interventions for people with traumatic stress and intimate partner violence; and providing group space for men to share their experiences with stress, problem solving, and coping.

Some participants preferred small group settings and some preferred individual therapy, while three participants indicated a need for both options to support all men with varying privacy concerns. Most men preferred in-person meetings, with the caveat that having the option to video conference could increase accessibility.

Suggestions for session length and frequency included an intensive format (four hours twice per week), weekly (an hour once a week), or monthly (once a month, biweekly for 1.5 h). Multiple participants highlighted how the length, frequency, and duration of therapy will depend on the individual, highlighting the overall theme of providing flexible, patient/client-centered options for therapy, group support, and social work and legal services.

## Discussion

Similar findings across quantitative and qualitative methods confirm that it is erroneous to consider LSMM with HIV a cultural monolith with universal experiences of minority stress or with similar needs regarding mental health and social services. LSMM with HIV vary in terms of sociodemographic characteristics and experiences of stigma, discrimination, and minority stress. There was within-person heterogeneity regarding the sub-constructs for stigma and discrimination, such as differences between forms of discrimination within an individual, differences between prior and current experiences of discrimination among participants who moved to the USA and experienced less stigma after immigrating, and differences among older participants who witnessed changes in discrimination over time. Surveys and interviews both indicated a variety of combinations of identities to which discrimination was directed (e.g., race, ethnicity, gender, weight, income, HIV status), which varied across participants. Interviews indicated that even participants who reported experiencing discrimination at some point in the last year experienced less anti-sexual minority discrimination in South Florida compared to other regions in the USA and in other countries. As would be expected by intersectional theories and supported by prior research, many indices of discrimination were worse among Latino men from minoritized racial groups [4, 25, 32].

In the survey, the highest discrimination was seen among men who had lived in the USA for fewer than 5 years. Interviews suggest higher discrimination among men in the USA for fewer than 5 years could be a result of discrimination in countries of origin or due to low acculturation, such as difficulties speaking English or disempowerment because of immigration status. There was also high discrimination among individuals born in the USA compared to men in the USA for 5 or more years, and this difference was most pronounced with past-year day-to-day discrimination; interviews suggest one possibility is that men who were born outside of the USA view the USA as less discriminatory in comparison to higher levels of discrimination in their country of origin. As with other stressors, discrimination is likely impacted by appraisal processes, with overall experiences influenced by both discriminatory experiences and personal appraisals of these experiences [33]. Together, the surveys and interviews highlight the compounding nature of discrimination among individuals with multiple marginalized characteristics, such as being a racial minority or a recent immigrant, as well as the context-dependent nature of intersectional discrimination.

Although experiences of discrimination were not universal, with 28% of individuals reporting they did not personally experience or witness someone else experiencing unfair treatment or discrimination in the past year, instances of overall discrimination and discrimination resulting in physical and sexual assault remain unacceptably high. The frequency of being threatened with a physical or sexual attack (36%), being physically attacked (36%), and being sexually assaulted (35%) is notable given the potential harm and risk for post-traumatic stress. As indicated in the interviews, participants attributed negative feelings about their sexual orientation to experiencing violence, and rejection after HIV status disclosure contributed to traumatic stress and avoidance. One encouraging finding was that participants indicated discrimination directed towards sexual orientation has decreased over time, but more work is needed to improve the safety of sexual minority men globally.

### Implications for Research, Practice, and Policy

The Health Stigma and Discrimination Framework provides a useful approach to conceptualize the fluid nature of stigma among individuals with HIV and other chronic health conditions [34]. This framework does not distinguish between “stigmatizer” versus “stigmatized” at the individual level to intentionally highlight the sociocultural factors that drive stigma [34]. This is relevant in the case of intersectional stigma in the Latino community, where experiences of discrimination are context dependent. For example, White Latino men who experienced discrimination based on sexual orientation in their country of origin may experience a decrease in sexual orientation discrimination after immigrating to the USA and have increased power in comparison to racial minority Latino men in the USA, but they may also experience new anti-Latino and anti-immigrant discrimination in certain settings. In Miami-Dade, Latinos are likely to be both the victims and perpetrators of anti-sexual minority hate crimes, highlighting the complexity of discrimination within Latino communities [35]. Using the Health Stigma and Discrimination Framework to interpret findings suggests that Latino SMM living with HIV should not be considered a universally “stigmatized” group, but policy interventions should focus on social, political, and economic drivers of stigma and discrimination, and individual-level interventions to mitigate harm should be provided to affected individuals [34].

Our findings show there is a need for English and Spanish-language mental health and coping interventions that are culturally adapted to address the high prevalence of violence that could result in trauma, while also being responsive to the heterogeneity of experiences and intervention preferences between Latino men. At a policy level, it is critical to continue ensuring funding for the Ryan White HIV/AIDS Program, which covers the costs of mental health services among people living with HIV who have a low income. Currently, undocumented individuals are eligible for Ryan White funding, and it is essential that these protections remain in place. The findings of this study have policy implications regarding the HIV treatment cascade and care continuum [36]. Because the participants were living with HIV, it is essential to leverage federal, state, and local policies and laws to ensure that discrimination and other mental health conditions do not interfere with retention in HIV care, adherence to antiretroviral therapy, and the achievement and maintenance of viral suppression to prevent disease progress and decrease the risk of HIV transmission [36]. This is also in alignment with the goals of U.S. Department of Health and Human Services’ plan to end the HIV epidemic by 2030 [37].

Our findings are aligned with prior research on hate crimes in Miami-Dade County, which show that 48% of sexual or gender minority Latino respondents experienced victimization in the past 5 years, with 30% reporting physical or sexual assault, and 95% reporting victimization due to sexual minority identity; however, only 15% of incidents were reported to the police [35]. Policy recommendations resulting from this research included establishing protocols for detecting hate crimes, developing a specialized workforce to identify and manage hate crimes, creating a dedicated victim support center, engaging with victims in lieu of subpoenas, providing training to criminal justice practitioners, modifying hate crime statutes to include gender identify as a protected class and include crimes that were partially motivated by hatred, conducting additional research, and engaging in communication and awareness building to support hate crime reporting [35]. Our findings are also aligned with existing policy positions of the American Psychological Association, including statements supporting humane immigration policies that consider the well-being of immigrants and refugees; the provision of mental health and social services to refugees and immigrants; and government-sponsored efforts to collect and publish statistics on hate crimes, provide services to victims of hate crimes, and develop interventions to eliminate hate crimes [38, 39].

With respect to individual-level interventions, increasing funding for the training and employment of Spanish-speaking mental health providers may help meet the need for greater Spanish-language trauma-informed mental health services. Perceived drivers of discrimination reported by men in our study included norms surrounding masculinity and religion, and these could be addressed in individual-level interventions focused on sexual minority stress. For example, participants felt pride in response to seeing Latino artists succeed, so it could be helpful to have greater representation of similar successes among LSMM and people living with HIV. While limits to discussion of sexual identity in educational settings could hinder this type of representation, this could be overcome using critical consciousness interventions, which have been used to provide education on gay historical figures to Black sexual minority youth living with HIV in HIV clinic settings and could be applied to LSMM with HIV [40].

While individual therapy is most easily tailored to an individual’s needs, most participants preferred group settings, which are also more cost effective and can range from formal group therapy to informal support groups. Interviews confirmed the need for trauma-focused care and a variety of intervention formats, including individual and small-group options and in-person and telehealth sessions, to meet the needs of LSMM with HIV. Participants noted that they often struggled with more urgent or impactful stressors than discrimination, such as interpersonal conflicts, caregiving, and financial stressors. Mental health interventions for LSMM with HIV may be more relevant if they address stress management broadly, while also providing support for specific stressors among Latino men, such as legal/immigration assistance. Interventions prioritizing sexual orientation as an identity may be viewed as less relevant to LSMM with HIV, whereas interventions tailored for Latino/Hispanic men who are negatively impacted by machismo, which is not limited to sexual minority men, may be more relevant [41].

LSMM with HIV who are from minoritized racial groups and who have recently moved to the USA had the highest discrimination and may benefit from additional support. Our recruitment efforts suggest studies to adapt interventions for these men will require dedicated time and resources to recruit sufficient samples of Black, Indigenous, and Multi-Racial Latino men, since they represent a smaller proportion of Latino individuals, and because individuals experiencing greater marginalization are typically underrepresented in the healthcare system. Recruitment in our study was facilitated by offering activities in Spanish (64% completed activities in Spanish), by using consent-to-contact databases maintained by National Institutes of Health-funded AIDS research centers, and through the existing integration of clinical and research-related HIV services at our university. Recruitment was hindered by not having weekend or after-hours options for study visits, which was done to process biospecimens in our on-site laboratory, but which limited participation of individuals who were busy during the traditional workday. Future studies prioritizing LSMM with HIV would benefit from more flexible scheduling for study visits, and developments in at-home/remote biomarker collection could mitigate scheduling difficulties. Additional recruitment barriers included the screening of men who were not eligible because they were not sexual minority individuals or were not living with HIV, but the decision was made to leave these details off the recruitment materials in conversation with the community advisory board, which emphasized the need for privacy. Lastly, many individuals did not answer their phones when called, so strategies to reduce the gap between screening and enrollment, such as screening in-person and immediately enrolling individuals in the study, or having alternative forms of contact, could support future study enrollment.

No participants discussed current events such as the “Don’t Say Gay” bill in their interviews, and there was little description of discrimination based on Latino ethnicity. Several explanations for the lack of discussion of the political climate are possible: (1) we did not ask about it specifically, (2) the large Latino population in Miami may insulate participants from experiencing anti-Latino/Hispanic discrimination, (3) participants may not view the discourse or laws as discriminatory or relevant to them, and (4) Latino individuals may avoid media as a coping strategy [21]. This study was conducted in a region with a large and diverse Latino population with a uniquely high number of individuals from Caribbean countries. The quantitative sample was representative of Miami-Dade in terms of sociodemographics [20], and is therefore likely to be generalizable to South Florida. Findings on the prevalence of violence in our sample were nearly identical to larger-scale research conducted in Miami-Dade, further supporting generalizability of findings to the area despite the smaller sample [35]. Research is needed to assess whether the findings are generalizable to different historical periods, political climates, and regions.

This study is limited due to the small sample size, which was not intended to be powered to identify causal effects, but to assist in the cultural tailoring of future research with this priority population. Combining Black, Indigenous, Multi-Racial, and other-identified into a single non-White category to represent historically minoritized groups may have obscured important differences between racial groups, and future research is needed with larger samples to perform multiple-group analyses. The triangulation of qualitative and quantitative methods strengthens this descriptive study, which is the first to provide detailed qualitative and quantitative insights regarding the diversity of intersectional discrimination, stigma, and minority stress experiences among LSMM with HIV in Miami in a quickly changing sociopolitical climate.

In conclusion, LSMM with HIV represent a diverse, multi-cultural community, and combining precision behavioral medicine and culturally informed approaches may be necessary to meet men’s distinct mental and physical health needs. Findings suggest one generalized intervention may not be as helpful as offering multiple options, including legal support, group and individual sessions, in-person and telehealth sessions, trauma-informed therapy, and tailored programs for newly diagnosed LSMM with HIV, racial minority LSMM, and men recently arriving to the USA. Research is needed to identify how to best match LSMM with HIV to the ideal intervention and how to offer multiple interventions in low-resource settings. This culturally informed, yet adaptable approach is necessary to decrease disparities along the HIV care continuum among Latino men in the USA.
